# Supplementary material for: Are carbon emissions trading and green financial instruments synergistic? -Comprehensive quantitative research based on content analysis
Source: PLoS One. 2024 Mar 7;19(3):e0298601. doi: 10.1371/journal.pone.0298601 (PMC10919601; doi:10.1371/journal.pone.0298601)
Supplement: S1 Data — (ZIP) [file pone.0298601.s001.zip › Data/地级市Z_F工作报告.zip!/─┌├╔╣┼/╢⌡╢√╢α╦╣╩╨/└┤╫╘╣1⁄2╓┌║┼í╛┬φ┐╦╩2╛▌═°í┐_2018.docx]

各位代表：

　　本届政府2013年1月就职，任期即将结束。现在，我代表市人民政府向大会报告工作，请予审议，并请市政协委员和列席人员提出意见。

　　　一、过去五年工作回顾

　　市三届人大一次会议以来的五年，鄂尔多斯走过了极不平凡的奋斗历程。五年来，面对经济下行压力加大、结构调整阵痛加剧、矛盾风险集中显现等一系列严峻挑战，我们在中央、自治区和市委坚强领导下，深入贯彻落实习近平总书记系列重要讲话和考察内蒙古重要讲话精神，积极应对挑战，奋力攻坚克难，全市各项事业发展取得新成效、迈出新步伐，为全面建成小康社会奠定了更加坚实的基础。

　　这五年，综合实力持续提升。地区生产总值年均增长7.9%，增速高于全国、全区平均水平；累计完成公共财政预算收入2122亿元，税收占比增加9个百分点，收入质量明显提高；完成固定资产投资1.4万亿元，年均增长11.5%，一大批产业提质、基础改善、民生保障类重大项目建成投用；社会消费品零售总额年均增长9.4%，达到3300亿元，消费升级步伐加快；城乡居民人均可支配收入达到43519元和16703元，年均分别增长8%和9.1%，群众生活更加殷实。主要经济指标全区领先，综合实力稳居中西部前列。

　　这五年，转型升级步履坚实。煤炭行业技术装备和安全生产水平持续提高，新增电力装机635万千瓦、煤化工产能1180万吨，煤炭就地转化率达到19%，成为国家4个现代煤化工产业示范区之一。新兴产业发展壮大，传统产业更新换代，非煤产业增加值占到规模以上工业增加值的50%，提高18.4个百分点。农牧业提质增效，粮食年产量突破30亿斤，牧业年度牲畜存栏量稳定在1300万头只左右。现代服务业快速发展，旅游人数突破千万人次，服务业占比达到42%，多点支撑、多元互补的现代产业体系正在形成，经济发展更具活力和韧性。

　　这五年，改革开放不断深化。经济、生态和社会事业领域共形成改革成果349个，在商事制度、智慧政务、自然资源审计、国地税合作等方面形成了一批“鄂尔多斯经验”。加强对外交流合作，国际航空口岸正式开放，呼包鄂协同发展迈出新步伐。深入实施人才鄂尔多斯战略，培养引进各类人才9.7万人；全面推进创新发展，建成各类科技创新载体54个，新增国家高新技术企业31家，市高新区升级为国家级高新区，获批国家农业科技园区，我市进入中国最具成长力创新型城市行列。

　　这五年，城乡环境品质倍升。中心城区一体化步伐加快，城市承载能力显著增强。康巴什区经国务院批准正式设立。特色城镇建设成效明显，10个乡镇被评为全国特色景观旅游名镇。美丽乡村建设深入实施，农村牧区人居环境和基本公共服务水平大幅提升。基础设施持续改善，新增高等级公路1100公里，呼包鄂、呼准鄂动车开行，鄂尔多斯机场迈入全国中型机场行列，供水、供电和通信网络保障能力全面增强。我市蝉联全国文明城市，鄂前旗、准旗被评为全国县级文明城市，建成中国西部首个文明城市群；荣获国家卫生城市、国家园林城市称号，被列为国家智慧城市试点。

　　这五年，生态建设成效显著。深入实施天然林保护、退耕还林、重点区域绿化等一批重点生态建设工程，完成林业建设712万亩、草原保护建设2251万亩、水土流失综合治理907万亩，森林覆盖率达到26.8%，地区生态环境显著改善，获国家森林城市、全国绿化模范城市等殊荣。资源节约型、环境友好型社会建设取得突破，全面实施大气、水、土壤污染防治计划，解决了一批突出环境问题，超额完成节能减排任务，空气优良率达到85%以上、位居全国前列，良好的生态环境已经成为鄂尔多斯新的亮丽名片。

　　这五年，民生福祉持续增进。脱贫攻坚取得决定性进展，国家标准线下贫困人口4.98万人实现稳定脱贫，贫困发生率由4.74%降至0.13%，基本消除绝对贫困。大众创业、万众创新蓬勃兴起，新增城镇就业15.7万人，新增市场主体5.6万户。社会保障体系更加健全，保障标准持续提高。建设保障房1.4万多套，改造棚户区6.1万多套。教育综合实力稳居全区前列，医药卫生体制改革全面深化，文体事业得到长足发展。社会治理体系进一步健全，社会大局和谐稳定，全市人民获得感、幸福感、安全感持续提升。

　　这五年，自身建设全面加强。深入开展党的群众路线教育实践活动、“三严三实”专题教育和“两学一做”学习教育，严格落实中央八项规定精神和国务院“约法三章”，标本兼治抓好中央、自治区党委巡视整改，政府系统作风明显改善。公车改革全面完成，市本级“三公”经费下降12%。深化“放管服”改革，四级政务服务体系基本建成，权责清单全部公布，精简优化行政权力事项1040项，审批时间缩短一半以上。主动接受人大依法监督和政协民主监督，严格依法行政，法治政府建设迈上新台阶。

　　各位代表，2017年是大事多、要事多、难事多的一年，我们全力抓重点、攻难点、创亮点，保持了全市经济社会稳中有进、稳中向好的发展势头。针对成因复杂、多年累积的部分经济数据虚增问题，下决心挤掉了水分，卸掉了发展包袱，为轻装上阵、扎实前进创造了良好条件。调减后，预计地区生产总值增长7%，公共财政预算收入增长5.1%，城乡居民人均可支配收入分别增长8.2%和7.9%。一年来，我们精准发力稳增长。深化供给侧结构性改革，出台促进工业稳定增长、金融支持实体经济、鼓励引导社会投资等一系列政策措施，完成煤炭去产能495万吨，消化房地产库存511万平方米，降低企业各类成本79亿元。实施亿元以上重点项目304项，全市完成固定资产投资3050亿元。纳林河、准格尔中部、新街台格庙等矿区总规获批；蒙西至天津南、上海庙至山东电力外送通道全线贯通，部分配套电源点开工建设；中天合创133万吨煤制烯烃、伊泰120万吨精细化学品等36个重点工业项目建成投产。一年来，我们坚定不移调结构。京东方二期、云泰互联、杭锦旗光伏发电站、久科环保产业园、中国联通云基地等项目进展顺利，新兴产业规模不断壮大。鄂前旗红色培训教育基地、康巴什康镇等旅游亮点项目投入运营，接待游客、旅游收入均增长20%以上。新引进金融机构2家，新增内蒙古股权交易中心挂牌企业62家。与天津物产集团共同组建的供应链公司投入运营，神华多式联运智慧物流园区开工建设。北京动物园批发市场入驻，康巴什乐康吧等新型商业项目建成。新培育农牧业龙头企业44家、公司化养殖园区10处、养殖大户300户，新增现代农牧业节水基地20万亩。一年来，我们蹄疾步稳抓改革。全面推行“多证合一、一照一码”制度，综合行政执法改革基本完成，市政务服务中心通过国家标准化试点验收。主动融入呼包鄂协同发展，与包头市共同被确定为国家首批资源型城市产业转型升级示范区。成立全市众创空间联盟，设立内蒙古煤基新材料研究院、中国生产力学院鄂尔多斯分院，新增院士工作站2家、自治区级众创空间17家、国家级小微企业创业创新示范基地2家。鄂尔多斯综合保税区获批并启动建设。一年来，我们全力以赴优环境。实施城市绿化提升工程112项，新增城区绿地570万平方米，达旗、准旗、鄂前旗被评为国家园林县城。加大基础设施建设力度，棋盘井至乌海、敖勒召其至东道梁等高速公路建成，沿河、陶鄂等铁路完工，鄂前旗通用机场开工建设，鄂尔多斯至北京双路由数据传输通道建成。大力整治环境突出问题，中央环保督察7项年度整改任务、棋盘井蒙西地区71项年度环境整治任务全面完成。一年来，我们倾心尽力惠民生。扶持市低收入人口2.1万人，国家标准线下贫困人口680人达到脱贫标准。新建、改建中小学、幼儿园16所，全市高考汉授、蒙授一本上线率创历史新高。8个旗区获评国家卫生县城，成为国家幸福家庭创建活动示范市。成功举办国际马拉松、国际文化创意大会等活动，被中国作协授予“中国诗歌之城”称号。成立全区首家青少年足球学院，承办全国幼儿足球交流大会。推进城乡居民基本养老、医疗保险制度并轨，城乡低保分别提标6%，农村特困人员集中和分散供养标准每人每年提高2000元和1884元。一年来，我们未雨绸缪防风险。多措并举化解金融风险，处置银行不良贷款80亿元；制定出台政府债务化解五年规划和年度方案，政府债务置换率达85.5%，完成政府债务化解年度目标；加强政银企合作，妥善解决了部分企业债券兑付危机。建成市旗两级安全生产信息化监管平台，安全生产事故死亡人数下降20.6%。推进食品安全城市创建，国家食品安全检测能力项目建成投用。大力开展信访积案百日攻坚行动，信访总量下降45.3%。加大清欠力度，农民工工资全部足额兑付到位。强化社会治安综合治理，深入开展缉枪治爆和打击“黄赌毒”违法犯罪专项行动，刑事案件下降16.1%。

　　2017年是自治区成立70周年，我们开展了形式多样的系列庆祝活动，民族团结进步事业不断加强，全市各族人民热爱家乡、建设家乡的热情更加高涨。我们成功举办《联合国防治荒漠化公约》第十三次缔约方大会和第六届库布其国际沙漠论坛，向世界贡献了荒漠化治理的鄂尔多斯智慧和模式，向世人展现了一个现代、文明、大美的鄂尔多斯。

　　习近平总书记向两个大会分别发来贺信，对我们的工作给予充分肯定，给全市人民带来极大的鼓舞和激励，凝聚起了做好鄂尔多斯工作的强大正能量！

　　各位代表，历尽天华成此景，人间万事出艰辛。

　　回首过去五年，走过的奋斗历程令人难忘。五年前，受宏观经济影响，能源产品价格下跌、房地产市场持续低迷、民间借贷矛盾集中爆发，我市经历了发展最为艰难的时期。但是，历经风雨、饱受挫折的鄂尔多斯，没有被困难压倒和打垮，经过全市上下五年艰苦努力，我们顶住了压力、经受了考验、战胜了挑战，经济社会发展的稳定性、协调性、可持续性进一步增强。今天的鄂尔多斯，以一个更加亮丽的姿态，再次站在了发展前沿，迈上了更高台阶，重塑了崭新形象！这些成绩的取得，是中央、自治区和市委正确领导、大力支持的结果，是历届班子继往开来、接力奋斗的结果，是全市广大干部群众攻坚克难、团结拼博的结果。在此，我代表市人民政府，向全市各族人民，向各位代表、委员，向各民主党派、工商联、人民团体，向驻市部队、武警官兵和公安干警，向所有关心、支持、参与鄂尔多斯改革发展的社会各界人士，致以崇高的敬意，表示衷心的感谢！

　　回首过去五年，积累的发展经验弥足珍贵。我们在实践中深刻体会到，推动鄂尔多斯持续健康发展，必须提高政治站位，树牢“四个意识”，坚决贯彻落实中央、自治区和市委决策部署，在全国、全区发展大局中找准自身战略定位，这样才能确保发展始终沿着正确方向前进；必须把发展放在首要位置，坚定发展信心，顺应发展大势，全面落实新发展理念，坚定不移推进转型发展，这样才能不断提高经济发展的质量和效益；必须发扬敢闯敢试、敢为人先的精神，保持改革创新的勇气、锐气和朝气，向改革要活力、向创新要效益、向开放要空间，这样才能持续增强地区自主发展能力；必须坚持人与自然和谐共生，处理好绿水青山与金山银山的关系，处理好当前利益与长远利益的关系，全面加大环境保护力度，这样才能实现更可持续的长久发展；必须坚持以人为本，切实保障和改善民生，解决好涉及老百姓切身利益的热点难点问题，让人民群众得到更多实惠，这样才能不断提高发展的向心力；必须全面从严治政，强化依法行政理念，加强党风廉政建设和反腐败斗争，驰而不息转政风，合力攻坚抓落实，这样才能为发展提供坚强的保障。

　　回首过去五年，存在的矛盾问题更需直面。我们清醒地看到，我市经济社会发展还存在不少困难，特别是发展不平衡、不充分的问题还很突出。经济发展质量效益不高、产业结构不优，传统资源型产业比重过高，战略性新兴产业刚刚起步，现代服务业发展还不充分，转变经济发展方式任重而道远；发展制约因素增多，资源环境约束趋紧，招商引资比较优势下降，产业性重大项目储备不足，固定资产投资增速放缓，稳增长的压力仍然较大；科技创新能力不强，人才结构性短缺明显，对外开放水平较低，一些改革举措没有真正落到实处，发展的内生动力还需进一步增强；生态环境依然脆弱，环保责任落实不到位，破坏生态现象还未从根本上杜绝，保护环境还需付出更大努力；基础设施保障能力有待提升，基本公共服务供给质量不高，人民群众在就业、教育、医疗、居住、养老等方面还面临不少难题，社会治理能力、社会文明程度还需进一步提高；城乡、区域发展还不平衡，特别是农村牧区发展相对滞后，农牧民增收乏力，脱贫攻坚越往后难度越大，全面建成小康社会还需付出更加艰苦的努力；金融风险不断累积、政府债务超过合理水平、安全生产形势复杂、信访事件仍然较多、社会治安压力加大，各种矛盾隐患和潜在风险交织叠加，维护社会稳定的任务还十分艰巨；政府职能转变不够到位，依法行政理念尚未牢固树立，经济数据不实、决策程序不规范、作风转变不到位等问题和干部不敢担当、为官不为、本领恐慌等现象不同程度存在，政府自身建设仍需进一步加强，等等。这些问题，必须高度重视，采取有力措施，认真加以解决。

　　　二、今后五年工作展望

　　党的十九大，绘就了决胜全面建成小康社会、夺取新时代中国特色社会主义伟大胜利的宏伟蓝图，必将凝聚起全民族逐梦前行的磅礴力量。伟大梦想鼓舞人心，伟大事业催人奋进。今后五年，是我们国家“两个一百年”奋斗目标的历史交汇期，也是鄂尔多斯大有作为的重要战略机遇期。我们坚信，只要始终保持清醒头脑，始终坚定发展信心，抢抓机遇，锐意进取，就一定能够谱写新时代鄂尔多斯发展更加辉煌的美好篇章!

　　今后五年政府工作的总体要求是：高举中国特色社会主义伟大旗帜，全面贯彻落实党的十九大精神，以习近平新时代中国特色社会主义思想为指导，统筹推进“五位一体”总体布局，协调推进“四个全面”战略布局，牢固树立和践行新发展理念，坚持以提高发展质量和效益为中心，突出抓重点、补短板、强弱项，打好防范化解重大风险、精准脱贫、污染防治三大攻坚战，坚守发展、民生、生态底线，更好满足人民日益增长的美好生活需要，确保全面建成小康社会，开启鄂尔多斯现代化建设新征程。

　　今后五年政府工作的目标是：全力推动经济高质量发展，总量保持全区领先，质量效益明显提升；转型发展取得新突破，创新能力显著增强，多元化中高端现代产业体系基本建立；城市宜居水平大幅提升，农村牧区振兴取得重要进展，城乡更具品质、更富魅力；环境质量持续改善，绿色发展方式基本形成，生态文明建设取得更大成效；民生社会事业全面进步，人民生活更宽裕，社会大局更和谐。

　　（一）加快转型升级，构筑现代产业新格局。坚持质量第一、效益为先，以供给侧结构性改革为主线，推动经济发展质量变革、效率变革、动力变革，加快构筑实体经济、科技创新、现代金融、人力资源协同发展的产业体系。提高资源综合利用率和精深加工度，将鄂尔多斯打造成中国重要清洁能源输出基地和煤化工产业基地。紧盯未来产业发展方向，着力在新产业、新模式、新业态上开疆拓土，推动互联网、大数据、人工智能和实体经济深度融合，大力发展绿色制造业，持续做大战略性新兴产业规模。加快发展现代服务业，建成国家全域旅游示范区和区域性金融中心、物流中心、会展中心。深化农牧业供给侧结构性改革，坚持走规模化、品牌化、绿色化发展道路，促进农村牧区一二三产融合互动，进一步提高农牧业现代化水平。

　　（二）全面改革开放，培育经济发展新动能。不断深化“放管服”改革，加强营商环境建设，让优质政府服务成为我市新的比较优势和核心竞争力。大力实施创新驱动发展战略，加快建立以企业为主体、市场为导向、产学研深度融合的技术创新体系，全面推进大众创业、万众创新，对接实施国家重大科技项目，争创国家自主创新示范区。着力引进培育高素质人力资源和各类实用型人才，健全科学合理的分配政策，让科研人员通过创新获得更多收益，让人才无后顾之忧、有施展空间。提升开放型经济水平，深度融入“一带一路”建设，积极申请建设自由贸易港，打造草原丝绸之路经济带和中俄蒙经济走廊重要节点城市，提高我市在国家向西开放格局中的战略地位。

　　（三）促进城乡融合，打造品质宜居新家园。完善市域空间布局，推进“多规合一”，构建以中心城区为核心、区域性中小城市为支撑、重点产业镇和特色小城镇为节点、新型村庄为补充的新型城镇化空间体系。全面提升城市建设管理水平，坚持精品化建设、园艺化打造、智慧化管理，增强城市功能，优化城市环境，提高城市品位，建设宜居宜业宜游品质城市。加大综合基础设施建设力度，不断提升基础设施承载保障能力。促进市域均衡协调发展，提高鄂前旗工业化水平，加大杭锦旗扶贫开发力度，支持达旗县域经济发展。统筹推进城乡公共服务均等化，实现常住人口全覆盖、同标准。全面实施乡村振兴战略，补齐农村牧区发展短板，从根本上改善农村牧区生产生活条件，让农村牧区产业更兴旺、生态更宜居、乡风更文明、治理更有效、生活更富裕。

　　（四）突出生态优先，厚植绿水青山新优势。牢固树立社会主义生态文明观，实施全市生态环境保护五年行动计划，创建国家生态文明先行示范区，建成国家环保模范城市，构筑起坚实的祖国北疆生态安全屏障鄂尔多斯防线。深入实施重大生态系统保护和修复工程，推进荒漠化、丘陵沟壑、水土流失、采煤塌陷区等综合治理，持续提升生态系统质量和稳定性。坚持走绿色发展之路，严守环境质量底线、生态保护红线、资源消耗上限，合理布局生产、生活、生态空间，倡导绿色低碳生活方式，形成人与自然和谐共生的发展格局。着力解决突出环境问题，加大水资源保护力度，开展土壤污染防治行动，坚决打赢蓝天保卫战。深化生态文明体制改革，健全生态文明建设制度体系。

　　（五）坚持共建共享，满足群众生活新需求。全面巩固提升脱贫攻坚成效，持续提高城乡居民收入水平。坚持就业优先战略，实施更加积极的就业政策，实现更高质量和更充分的就业。加快推进教育现代化，办好人民满意的教育。全力打造健康鄂尔多斯，努力为人民提供全方位、全周期健康保障。加大文化惠民力度，不断丰富群众精神文化生活。完善社会保障体系，建成覆盖全民、城乡统筹、权责清晰、保障适度、可持续的多层次社会保障体系。高度重视风险防范化解工作，突出抓好政府债务化解，确保政府债务率降至合理水平。创新社会治理模式，完善社会治理体系，维护社会大局和谐稳定。创建全国民族团结进步示范市，巩固各民族共同团结奋斗、共同繁荣发展大好局面。

　　　三、2018年工作安排

　　今年是全面贯彻落实党的十九大精神开局之年，是改革开放40周年，也是新一届政府开篇布局、担当作为的起步之年。做好今年工作，意义重大。统筹考虑，确定主要预期目标为：地区生产总值增长6.5%左右，城乡居民人均可支配收入分别增长7.5%左右，城镇登记失业率控制在3.1%以内，供给侧结构性改革取得实质性进展，完成节能减排目标任务，财政金融风险有效防控，化解政府债务完成年度任务。

　　围绕上述目标，重点做好八个方面工作。

　　（一）强化供需共同发力，着力保持经济平稳增长。

　　持续扩大有效投资。实施亿元以上重大项目233项，积极争取华星煤制气、新蒙煤制气、上海庙至山东电力外送通道配套新能源规划等获批，推动神华煤直接液化二三线、国电投烯烃、鄂安沧输气管道等工业项目建设；实施服务业重点项目100项，完成投资80亿元以上，保持服务业投资快速增长；实施东乌与包西联络线等12项重点铁路和包茂高速四改八、乌审旗至鄂旗等14项重点公路项目，开工建设杭锦旗等通用机场，实施红庆河、乌审500千伏输变电和城市配电网等工程，推进黄河鄂尔多斯段二期防洪、西北节水增效、考考什纳水库除险加固等重点水利项目。有效激发民间投资，支持民营资本依法进入更多领域，促进民间投资合理增长。

　　提高工业供给质量。启动提高煤炭行业综合竞争力三年行动计划，支持大型煤炭企业释放先进产能，煤炭产销量稳定在6亿吨以上。加快电力通道配套电源点建设，新增火电装机35万千瓦。开工建设神华粉煤灰提取氧化铝项目，确保蒙泰铝板带等项目建成投产。加快伊泰200万吨煤制油、北控40亿立方米煤制气、久泰60万吨甲醇制烯烃等项目建设，确保新能稳定轻烃、易高合成气制乙二醇等项目建成投产，新增高端煤化工产能110万吨。加强与中石化等企业在天然气勘探开发领域合作，稳步提高天然气产量，大力发展CNG、LNG产业。深入实施羊绒振兴计划，保护好“鄂尔多斯”民族品牌。促进PVC、冶金、陶瓷、水泥等产业改造升级，开发塑料纤维、特种钢材、礼品陶瓷、新型建材等高端产品，引导传统产业创新品种、提升品质、培育品牌，提高科技含量和市场竞争力。

　　发挥消费基础作用。适应大众消费多样化、个性化需求，加快共享经济、健康养老、信息服务、教育培训、创意设计、健身休闲等新型消费业态发展，积极培育新的消费热点，建设国家文化消费试点城市。启动建设万达城市广场，提升沃尔玛、王府井、呼能广场等大型综合商业体发展水平。促进商贸流通业线上线下融合互动，打造一批新型智能化商圈。深入实施商标品牌战略，开展质量提升行动。加快城乡流通网络建设，深化供销社综合改革，挖掘农村牧区消费潜力。健全产品质量追溯体系，坚决打击制售假冒伪劣产品行为，让老百姓便捷消费、放心消费。

　　（二）全面振兴实体经济，着力提高经济发展质量。

　　加大科技创新力度。围绕创新型企业培育引进、创新平台建设、关键技术与创新产品开发三大任务，聚焦重点领域，加大科技创新力度，推动创建呼包鄂国家自主创新示范区。支持市高新区发展，引导鼓励各类创新资源、研发机构和高新技术项目向园区集聚，打造创新发展主阵地。认真落实人才发展和科技创新政策措施，大力引进、培育急需紧缺人才和创新创业团队，推进市科技领军人才驱动中心建设。激励知识产权创造，加强知识产权保护，弘扬劳模精神和工匠精神，营造全社会崇尚劳动、鼓励创新的良好氛围，让各类要素更多地向实业聚集。

　　培育新兴产业集群。积极申报“中国制造2025”试点示范城市，加快奇瑞、华泰新能源汽车和海明堡直升机等制造业项目建设。推动京东、世纪互联等大数据项目落地，全市物理服务器装机能力达到10万台以上，尽快将我市建成中国北方重要的数据储存、灾备中心。依托电力外送通道建设，实施库布其沙漠光伏应用基地等项目，新增新能源装机52万千瓦。开工建设尚德抗癌药物研发和中试基地。推进巨无霸锂电池、华明正极材料、新能石墨烯、中铁碳纤维等项目建设，抢占新材料产业发展先机。

　　打造良好营商环境。全面推行“一站办好、全市通办、最多跑一趟” 改革，推动政务服务全程网上办理，推行投资核准联评联审、联合验收等模式，激发各类市场主体活力。扩大“双随机、一公开”监管范围，推广信用监管和后评估模式。落实和完善支持实体经济发展政策措施，全面规范清理各类涉企收费，加大对乱收费的查处整治力度。拓宽企业融资渠道，推进多层次资本市场建设，扩大直接融资比例，增强金融服务实体经济能力。发挥政府资金引导作用，设立总规模100亿元的产业发展基金，集中扶持实体经济发展。制定园区振兴计划，突出优势、精准定位、差异化扶持，提高园区集约化发展水平，让园区成为实体经济发展的主要载体。

　　各位代表！企业是推动经济发展的主要力量，企业家是最难得的宝贵资源。我们要大力弘扬和保护企业家精神，构建“亲”“清”新型政商关系，亲而有度，清而有为，全力为民营经济发展创造更加宽松的条件，让鄂尔多斯非公经济再出发、企业家再出发，为振兴实体经济、促进地区发展作出新的更大贡献！

　　（三）做大做强现代服务业，着力培育发展新动能。

　　壮大文旅产业规模。充分挖掘民族文化和地方文化资源，全面推进文化旅游融合发展，加快蒙古源流、鄂尔多斯海洋馆、黄河大峡谷等项目建设。构筑“大成陵”旅游格局，拓展鄂前旗红色培训教育基地功能，发挥影视产业后发优势，打响“国际蒙元文化体验旅游首选地、国家西部红色旅游示范区、全域影视拍摄基地”品牌。大力发展乡村旅游，新建高品质旅游示范户100家、休闲观光农牧业庄园20家。完善配套服务设施，培育一批高端酒店、精品民宿和娱乐街区，打通便捷的旅游公交网络，增加热点城市旅游航线，建成全市游客集散中心，新建改造一批高标准旅游厕所。做大做强市文旅集团，发挥国有资本引领带动作用。开展“满意鄂尔多斯游”创建活动，提升旅游业服务水平。全年旅游接待人数、旅游收入分别增长18%和16%。

　　提升物流产业层次。出台优惠政策，积极搭建煤炭、天然气、煤化工、硅铁、PVC等大宗商品交易平台，扩大网上交易量，加快建设国家西部能源交易中心。完善规划，在重点铁路沿线建设一批现代化煤炭物流集装站，大力发展公铁、铁海联运新模式，提高煤炭外运能力。加快综合保税区基础设施建设，启动空港货运综合枢纽项目，建设地区快递集散中心、航空物流中心，将空港园区打造成全市物流业发展排头兵。引入实施货车帮等智慧物流项目，全面提升物流业信息化水平。申报国家跨境电子商务综合试验区，集聚电商企业，推动形成“大电商”格局。

　　完善金融产业体系。积极引进内蒙古银行、渤海银行以及保险、证券等金融机构入驻，鼓励符合条件的大型企业发起设立民营银行、企业财务公司和基金公司，实现各类金融业态全覆盖。支持地方商业银行增资扩股，推动准煤联社、伊旗矿区联社等6家信用联社改制为农商行。做大市投资担保公司，组建市金融控股集团公司。设立企业上市奖励资金，支持企业股改和上市挂牌，新增主板上市企业1家、“新三板”挂牌企业2家、区域性股权市场挂牌企业30家。

　　（四）统筹推进城乡建设，着力提高城乡宜居水平。

　　深入开展城市品质提升行动。突出地域和民族特色，高起点、高标准做好城市规划，启动城市总规修编工作。完善城市功能，实施市政基础设施项目177项，新建城区供气管网370公里，完成旗区生活污水处理厂提标改造，引进垃圾无害化处理新技术、新工艺、新项目。推进东阿康一体化协同发展，实现基础设施互联互通、公共设施共建共享。提升园林绿化品位，新建和改造绿地800万平方米。实施东西红海子湿地恢复工程，完善水岸新城配套服务设施，提高乌兰木伦河南岸区域宜居水平。推进老旧小区改造，推行物业标准化管理模式。支持改善性住房需求，发展住房租赁市场，推进商业地产去库存，改造城市棚户区5399套，消化房地产库存240万平方米。推进“国家智慧城市”试点建设任务，实现中心城区数字化管理全覆盖。开展生态宜居县城创建工作，力争所有旗区达到创建标准。精心承办好鄂尔多斯国际那达慕大会、国际文化创意大会和羊绒、煤炭、文化产业博览交易会，着力打造西部地区特色会展城市，不断积聚商气人气。

　　全面实施乡村振兴战略。科学制定乡村振兴战略规划，确保开好局、起好步。深化农村牧区综合改革，完成集体土地确权颁证和房地一体登记工作，推进承包地“三权”分置，保持土地草牧场承包关系长期稳定。坚持质量兴农、绿色兴农，优化种养殖业结构，扩大绿色、有机、无公害农畜产品供给。培育壮大新型农牧业经营主体，加快农畜产品精深加工，新增产业化龙头企业20家、农牧民专业合作社300家，建设一批农村牧区电商示范户，让优质农畜产品打开市场、卖出好价格。加大耕地保护力度，深化粮食收储制度改革，解决农牧民“卖粮难”问题。大力培养农村牧区实用型、技能型人才。完善农牧业科技推广服务体系，提高农牧业机械化水平。启动农村牧区环境综合整治行动计划，推开乡镇垃圾、污水无害化处理试点，大力推广天然气、太阳能等清洁能源。深入开展乡风文明大行动，提升农牧民文明素质，传承优秀乡土文化，反对陈规陋习，弘扬时代新风，营造农牧民精神家园。

　　各位代表！农村牧区发展滞后，是我市全面建成小康社会的最大“短板”。我们将借助国家乡村振兴战略的东风，持续加大投入，全面改善生产生活条件，推动农村牧区全面振兴，让广大农牧民过上更有品质的好日子！

　　（五）深入推进改革开放，着力增强发展动力活力。

　　在提高改革实效上下功夫。深入推进供给侧结构性改革，抓好“三去一降一补”任务落实。深化综合行政执法体制改革，调整优化管理职能，健全联合执法机制，推动执法重心下移，加大对草牧场破坏、开荒种地、农村牧区环境污染等行为打击力度，把该管的事情管好。改革国有资本授权经营体制，优化国有资本布局结构，发展混合所有制经济，做大做强做优国有企业。规范公共资源交易行为，实现公共资源交易全方位标准化管理。加快农村牧区金融改革，全面推广“两权”抵押贷款模式。深化财税体制改革，加强全市综合治税平台建设，推行个体和零散税收社会化管理模式。完善户籍制度改革，促进农牧业转移人口市民化。开展增量配电试点业务，扩大电力多边交易规模，着力打造电力成本“洼地”。

　　在扩大对外开放上下功夫。加强与以色列等“一带一路”沿线国家合作交流，积极融入“中俄蒙经济走廊”建设，办好第二届中蒙博览会、中俄蒙国际青少年运动会等活动。提升与长江经济带、珠三角、晋陕宁等地区的合作水平，认真落实呼包鄂协同发展规划纲要，积极对接京津冀协同发展，加大与天津滨海新区合作力度。推动加工贸易发展，推广应用国际贸易“单一窗口”，促进贸易便利化，提升鄂尔多斯口岸管理水平。巩固和拓展国际航线，建设呼包鄂榆地区国际航空客货运中心。

　　在提升招商引资精准度上下功夫。创新招商引资模式，依托我市煤化工等优势产业，发挥规模集聚效应，大力开展产业链招商，吸引更多关联性、聚集性强的补链、延链项目落地；发挥资源优势，顺应资源市场配置开展招商，大力引进新能源、天然气加工、云计算大数据、节能环保等具有明显成本优势的项目；通过国资参股、企业并购、联合投资等形式，合作建设一批成长性好的项目。更加注重招商引资质量，重点引进技术新、效益好、带动性强的企业和产业，实现从“招商引资”向“择商选资”转变。

　　（六）持续巩固生态优势，着力提升生态文明水平。

　　实施好重点生态工程。开展大规模国土绿化行动，引导企业、社会组织、个人等各方面资金投入，持续加大生态建设力度。深入推进天然林保护、退耕还林、京津风沙源治理、十大孔兑生态减沙等国家重点生态工程，加快建设“四带”工程，启动实施呼包鄂沿黄生态走廊建设工程，完成林业生态建设150万亩、草原保护建设350万亩、水土流失综合治理180万亩。坚持保护优先、自然恢复为主，严格执行禁牧休牧轮牧和“草畜平衡”政策，落实好“三区”发展规划。精心筹办好中国鄂尔多斯国际沙产业博览会。

　　解决好突出环境问题。全力推进自然保护区内开发建设活动清理整治工作，完成棋盘井蒙西地区环境综合整治年度任务。加快绿色矿山建设，完成矿区环境综合治理66平方公里。强化水污染防治责任，全面落实河长制、湖长制，确保乌兰木伦河、龙王沟等主要河流水质稳定达标、城镇集中饮用水源地水质全部达标。推进土壤污染防治行动，完成全市农业用地土壤详查工作，深入开展化肥、农药减量化行动，提高畜禽养殖废弃物和农作物秸秆综合利用率。认真落实大气污染防治行动计划，健全空气质量监测预警体系，着力解决工业排放、燃煤取暖、粉尘污染等突出问题。

　　构筑好生态保护机制。全面推行自然资源资产负债表、领导干部自然资源资产离任审计和责任终身追究等制度，严格落实自然资源有偿使用、生态环境损害赔偿等制度，让制度真正发挥震慑作用。建立环境风险预测预警体系，强化企事业单位环境风险防范主体责任。加强与自治区环保投资集团合作，与中化集团共同组建环保产业研究院公司。加强环境执法监测体系和能力建设，完善信息化监管系统，健全联合执法机制，以零容忍态度，严厉打击环境违法行为。

　　各位代表！生态是鄂尔多斯最宝贵的财富、最重要的品牌。我们将牢固树立“绿水青山就是金山银山”的理念，尽最大努力保护好我们赖以生存的这片土地，为子孙后代留下更多的生态财富，为保护生态环境作出我们这代人的努力！

　　（七）加大公共服务供给，着力增强人民群众获得感。

　　实现更加充分的就业创业。实施积极就业政策，统筹做好各类群体就业工作，新增城镇就业2.9万人。推动创业带动就业，设立创业就业发展专项基金，打造一批专业化的众创空间，鼓励农牧民返乡创业。开展精准帮扶，确保零就业家庭动态清零。落实干部职工工资定期调整制度。实施农牧民增收行动计划，努力增加农牧民财产性、经营性、政策性收入。

　　构筑更加健全的社保体系。实施全民参保登记计划，建成准确完整的全民参保数据库，建设社保信息共享平台。推进医疗付费制度改革，开展按病种付费和综合性付费试点。深化医疗保险盟市级统筹工作，推动异地就医直接结算。强化社会救助兜底保障功能，城乡低保标准分别提高8%和8.5%，农村特困人员集中和分散供养标准分别提高10%和8.5%。加强困难群体救助，保障妇女儿童、老年人、残疾人合法权益，确保弱有所扶。

　　打造更高质量的优质教育。新建、改扩建中小学25所、幼儿园18所，全面完成农村牧区教师周转房建设任务。推进义务教育优质均衡发展，推动中心城区优质教育资源共建共享，着力解决“择校热”“大班额”、中小学生课外负担重等突出问题。提升教育信息化水平，建设全市“教育云”。办好学前教育，建立完善服务保障体系，解决好婴幼儿照护和儿童早期教育服务问题，确保幼有所育。构建现代职业教育体系，强化产教融合、校企合作。提升高等教育办学水平，打造一批知名专业和学科。优先发展民族教育，实施蒙古语授课教师素养提升工程，优化牧区学校布局。保障特殊群体平等接受教育。推进教师管理体制改革，完善教师薪酬增长机制。

　　提供更高水平的健康服务。启动杭锦旗国家卫生县城创建工作，加快实现全市国家卫生县城全覆盖。全面推开医联体、医共体和远程医疗建设，扩大家庭医生签约覆盖面，提升基层医疗卫生机构服务能力，构建分级诊疗新秩序。加快市第二医院、第四医院、中医医院住院楼等项目建设改造，进一步改善医疗卫生基础条件。完善全科医生培养、激励机制，大力引进高端医疗人才和知名专科。启动智慧卫生工程建设，提高全市医疗卫生信息共享和医疗协同水平。加快蒙中医药事业发展，打造蒙中医药健康旅游示范基地。大力发展健康产业，鼓励社会资本进入养老、医疗等领域，推广医养结合、居家养老和旅居候鸟式养老等服务模式。强化重大疾病防控，倡导健康文明生活方式，提升公众健康素养，积极创建健康城市。提升计生服务水平，提高出生人口质量。

　　发展更加惠民的文体事业。深入实施“十个一”文化工程，巩固国家公共文化服务体系示范区创建成果。支持文化展示、文化演示、文化市场发展，办好诗歌那达慕、草原国际舞蹈嘉年华、鄂尔多斯文化神州行、“一带一路上的乌兰牧骑”巡演等品牌活动，让鄂尔多斯文化走向全国、走向世界。积极推进“成吉思汗祭祀文化”申报世界非物质文化遗产，加大长城、萨拉乌苏等遗址保护力度。弘扬乌兰牧骑精神，加强乌兰牧骑建设，更好地发挥草原“红色文艺轻骑兵”作用。实施市民体质提升工程，推进全民健身公共服务体系建设，实现大中型体育场馆免费或低收费开放、所有嘎查村全民健身设施全覆盖。推进足球改革发展，建成青少年足球训练基地。办好国际马拉松等赛事活动，备战全区第十四届运动会、第五届残运会，争创好成绩。

　　坚决打赢脱贫攻坚战。统筹整合财政资金、扶贫贷款和社会资金，持续加大脱贫攻坚投入力度。发展壮大村集体经济，突出抓好产业扶贫，推行特色农牧业扶贫、光伏扶贫、旅游扶贫和电商扶贫等新模式。更加注重健康扶贫，提高贫困人口大病报销比例，切实解决群众因灾致贫、因病返贫问题。坚持扶贫与扶志、扶智相结合，激发贫困人口内生动力。

　　各位代表！人民对美好生活的向往，就是我们的奋斗目标。我们将加快社会事业发展，解决好群众最关心最直接最现实的利益问题，让全市人民生活得更舒心、更安心、更顺心！我们将以更大力度推进精准脱贫，确保小康路上，不让一个人掉队！

　　（八）有效防范化解风险，着力维护社会安全稳定。

　　全力化解财政金融风险。抓好政府债务管理，严控债务增量，规范政府举债融资、基金运作和购买公共服务等行为，确保债务“零增长”。健全政府投资项目监管体系，坚决制止违法违规融资担保行为，坚决叫停过度超前、盲目举债项目。有效盘活存量资产，多方整合国有资源，积极筹措化债资金。健全债务风险预警和应急处置机制，设立政府防范财政金融风险基金。加快处置化解银行不良贷款，降低贷款不良率，统筹解决好企业债券兑付等问题。加快非法集资涉案资产处置进度，严厉打击“老赖”行为。完善地方金融监管体制，严厉打击乱办金融、非法集资等违法违规金融活动，牢牢守住不发生系统性金融风险的底线。

　　全力做好安全生产工作。牢固树立安全红线意识，严格落实安全生产责任制，强化企业主体责任，积极推进企业安全生产标准化建设，广泛开展安全生产宣传教育培训。用法治手段解决安全生产问题，通过对违法行为的严惩，达到“企业不敢出事故、出不起事故”的效果。加强安全生产监管能力和信息化建设，扩大在线监管覆盖面。大力推进重点行业领域安全生产隐患排查和专项整治，确保全市安全生产形势持续稳定。

　　全力创新社会治理体系。强化社会治安管理，完善信息化、立体化社会治安防控体系，加大道路交通、消防等领域监管力度，提高反恐意识和能力建设，严厉打击各类违法犯罪活动。加大矛盾纠纷排查化解力度，有效化解信访积案。建立全市应急调度指挥系统，提升应急处置能力。构建农民工工资兑付长效机制，切实维护农民工合法权益。加强社区治理体系建设，发挥社会组织参与社会治理和提供公共服务的积极作用。加大普法力度，全面完善四级公共法律服务体系。落实“四个最严”要求，创建食品药品安全示范城市。践行社会主义核心价值观，深化群众性精神文明创建活动,提高市民素质和社会文明程度。积极推动军民融合深度发展，做好国防动员工作，巩固双拥模范城市创建成果。统筹做好国家安全、防震减灾、气象预警、民族宗教、外事侨务、人民防空、史志档案等各项工作。

　　各位代表！我们要清醒地认识到，前进的道路并不平坦，多年积累的各种风险隐患需要我们冷静面对、有效处置。我们将始终坚持稳中求进的工作总基调，保持战略定力，坚持底线思维，坚决打好风险防范攻坚战，确保鄂尔多斯经济发展行稳致远！

　　四、加强政府自身建设

　　立足新时代、谋求新发展、实现新目标，各级政府责任重大。我们要始终把为民、务实、清廉作为价值追求，全力打造人民满意的服务型政府。

　　牢记使命，忠诚履职。树牢“四个意识”，坚定 “四个自信”，坚决维护习近平总书记核心地位、维护党中央权威和集中统一领导，做到思想上对标对表、行动上紧跟紧随、执行上坚定坚决。严守政治纪律、政治规矩，决不搞上有政策、下有对策，不做表面文章、不折腾不懈怠，坚决贯彻中央大政方针和自治区、市委决策部署，做到政令畅通、令行禁止。履行好政府党组管党治党责任，推进“两学一做”学习教育常态化制度化，深入开展“不忘初心、牢记使命”主题教育，以党的建设新成效推动政府自身建设再上新水平。

　　厉行法治，依法行政。坚持法治思维和法治方式，把法治理念贯穿政府工作全过程，全面推行清单管理制度，做到职权法定、用权依法、法外无权，始终让政府权力在法治轨道上运行。自觉接受人大、政协监督，主动回应社会监督，大力推进政务公开，让公开透明成为政府工作的常态。科学编制政府立法计划，推动重点领域立法。严格执行重大决策程序，健全法律顾问制度和专家决策咨询制度，加强新型智库建设，提升政府决策水平。强化诚信建设，政府要带头讲诚信，做到言出必行、有诺必践，新官要理旧账，全面提升政府公信力。

　　改进作风，务实施政。坚持谋实事、出实招、求实效，大力弘扬立说立行、马上就办的作风，勇于攻坚克难，以钉钉子精神做实做细做好各项工作。深入实施“互联网+政务”行动，推进全市政务信息系统整合，实现数据资源开放共享。大兴调查研究之风，找准短板弱项，着力解决老百姓关注的热点问题。牢固树立长期过紧日子思想，厉行节约，反对浪费。旗帜鲜明鼓励担当者、保护改革者、宽容失误者，为干部大胆创新探索撑腰鼓劲。健全重点工作督查机制，坚决整肃庸政懒政怠政现象，推进追责问责制度化、常态化。

　　遵规守纪，廉洁从政。深入贯彻中央八项规定实施细则精神和自治区党委、市委实施办法，坚决防止“四风”反弹，严肃整治推诿扯皮、吃拿卡要、数据造假、有令不行等突出问题，全面推进作风建设从“宽松软虚”走向“严紧硬实”。加强审计监督，推进审计制度改革，实现公共资金、国有资产、经济责任审计等全覆盖。深化监察体制改革，健全监督体系。加强公共资金监管，严控“三公”经费等一般性支出。严格落实党风廉政建设主体责任，严肃查处各类违纪违法行为，始终保持遏制腐败高压态势。

　　各位代表，幸福都是奋斗出来的。让我们高举习近平新时代中国特色社会主义思想伟大旗帜，更加紧密地团结在以习近平同志为核心的党中央周围，在自治区和市委坚强领导下，不忘初心、牢记使命，守望相助、一往无前，为“建设亮丽内蒙古、共圆伟大中国梦”作出新的更大贡献！
